# Bayesian fractional polynomial approach to quantile regression and variable selection with application in the analysis of blood pressure among US adults

**DOI:** 10.1080/02664763.2024.2359526

**Published:** 2024-05-30

**Authors:** Sanna Soomro, Keming Yu

**Affiliations:** Department of Mathematics, Brunel University London, Uxbridge, UK

**Keywords:** Bayesian inference, fractional polynomials, non-linear quantile regression, quantile regression, parametric regression, variable selection

## Abstract

Although the fractional polynomials (FPs) can act as a concise and accurate formula for examining smooth relationships between response and predictors, modelling conditional mean functions observes the partial view of a distribution of response variable, as distributions of many response variables such as blood pressure (BP) measures are typically skew. Conditional quantile functions with FPs provide a comprehensive relationship between the response variable and its predictors, such as median and extremely high-BP measures that may be often required in practical data analysis generally. To the best of our knowledge, this is new in the literature. Therefore, in this article, we develop and employ Bayesian variable selection with quantile-dependent prior for the FP model to propose a Bayesian variable selection with parametric non-linear quantile regression model. The objective is to examine a non-linear relationship between BP measures and their risk factors across median and upper quantile levels using data extracted from the 2007 to 2008 National Health and Nutrition Examination Survey (NHANES). The variable selection in the model analysis identified that the non-linear terms of continuous variables (body mass index, age), and categorical variables (ethnicity, gender, and marital status) were selected as important predictors in the model across all quantile levels.

## 1. Introduction

Over the past three decades, the number of adults aged 30-79 with hypertension has increased from 648 million to 1.278 billion globally [65]. Hypertension is a highly prevalent chronic medical condition and a strong modifiable risk factor for cardiovascular disease (CVD), as it attributes to more than 45% of CVD and 51% of stroke deaths [56]. The risk of CVD in individuals rises sharply with increasing BP [10,16,20,38,39].

Continuous BP measurement has proven to be one of effective incident prevention. This implies that BP is the essential physiological indicator of the human body. When the heart beats, it pumps blood to the arteries resulting in changes in BP during the process. When the heart contracts, BP in the vessels reaches its maximum, which is known as systolic BP (SBP). When the heart rests, BP reduces to its minimum, which is known as diastolic BP (DBP).

Linear regression and polynomial regression analyses have been used in assessing the association between BP and risk factors contributing to various diseases [28,35,59]. It is evident that the polynomial regression models fit the data accurately in some research studies due to its adaptability of non-linearity property, yet face high-order polynomial approximation. The fractional polynomials (FPs), proposed by Royston and Altman [44], act as a concise and accurate formulae for examining smooth relationships between response and predictors, and a compromise between precision and generalisability. The FPs are parametric in nature and then intuitive for the interpretation of the analysis results. The FP approach has clearly established a role in the non-linear parametric methodology, especially with application by clinicians from various research fields, such as obstetrics and gynaecology [52], gene expression studies in clinical genetics [50] and cognitive function of children [46], and other medical applications, see [21,40,55], and amongst others.

However, modelling conditional mean functions observes the partial view of a distribution of response variable, as the distributions of many response variables such as the BP measures are typically skew. Then, ‘average’ BP may link to CVD, yet extremely high BP could explore CVD insight deeply and precisely. So, existing mean-based FP approaches for modelling the relationship between factors and BP cannot answer key questions in need. It is attractive to model conditional quantile functions with FPs that accommodate skewness readily. Quantile regression, introduced by Koenker and Bassett [27], provides comprehensive relationships between the response variable and its predictors, which are useful for median and extremely high BP measures in practical data analysis generally.

Zhan et al. [64] suggested quantile regression with FP as a suitable approach for an application, such as age-specific reference values of discrete scales, in terms of model consistency, computational cost and robustness. This approach is also used to derive reference curves and reference intervals in several applications [7,8,11,12,15,30,36], and amongst others, which allow quantiles to be estimated as a function of predictors without requiring parametric distributional assumptions. This is essential for data that do not assume normality, linearity and constant variance. Recently, reasonable amount of non-linear quantile regression analyses have been conducted in medical data analysis, see [25,37,57], and amongst others.

However, Bayesian approach to quantile regression has advantages over the frequentist approach, as it can lead to exact inference in estimating the influence of risk factors on the upper quantiles of the conditional distribution of BP compared to the asymptotic inference of the frequentist approach [63]. It also provides estimation that incorporates parameter uncertainty fully [62,63]. Some comparison studies have been conducted for both Bayesian and frequentist approaches, such as the analysis of risk factors for female CVD patients in Malaysia [26] and the analysis of risk factors of hypertension in South Africa [31]. The former revealed that the Bayesian approach has smaller standard errors than that of the frequentist approach. The latter also revealed that credible intervals of the Bayesian approach are narrower than confidence intervals of the frequentist approach. These findings suggested that the Bayesian approach provides more precise estimates than the frequentist approach.

Variable selection in Bayesian quantile regression has been widely studied in the literature, see [1–4,14,17,33], and amongst others. It plays an important role in building a multiple regression model, provides regularisation for good estimation of effects, and identifies important variables. Sabanés Bové and Held [47] combined variable selection and 'parsimonious parametric modelling' of Royston and Altman [44] to formulate a Bayesian multivariate FP model with variable selection that efficiently selects best-fitted FP model via stochastic search algorithm. However, in the present, no research studies have been conducted for variable selection in Bayesian parametric non-linear quantile regression for medical application, even though there is a limited amount of studies in case of non-regularised models, such as mixed effect models [53,60].

Therefore, in this paper, we explore a new quantile regression model using FPs and employ Bayesian variable selection with quantile-dependent prior for a more accurate representation of the risk factors on BP measures. The three-stage computational scheme of Dao et al. [17] is employed as a variable selection method due to its fast convergence rate, low approximation error and guaranteed posterior consistency under model misspecification. So, we propose a Bayesian variable selection with non-linear quantile regression model to assess how body mass index (BMI) amongst United States (US) adults influences BP measures, including SBP and DBP. The objective of this paper is to examine non-linear relationships between BP measures and their risk factors across median and upper quantile levels. The dataset used in this paper is the 2007–2008 National Health and Nutrition Examination Survey (NHANES), including the information on BP measurements, body measures and socio-demographic questionnaires.

The remainder of this paper is as follows. Section 2 presents the concept of FPs [44], quantile regression [27] and Bayesian variable selection with quantile-dependent prior [17]. The details of the NHANES 2007–2008 dataset used for the analysis are provided in Section 3. Section 4 applies the proposed method to the analysis, performs comparative analysis with two quantile regression methods and provides all the findings. Section 5 concludes this paper.

## 2. Methodology

Regression analysis is a technique that quantifies the relationship between a response variable and predictors. Quantile regression is a method to estimate the quantiles of a conditional distribution of a response variable and as such, it permits a more complete portrayal of the relationship between the response variable and predictors.

### 2.1. Quantile regression

Let *τ* be the proportion of a sample having data points below the quantile level *τ*. Given a dataset, $\{x_{i},y_{i}{\}}_{i=1}^{n}$ and the fixed quantile level *τ*, the $\tau^{\mathrm{th}}$ quantile regression model is represented as follows:

(1) $$\begin{array}{r} y_{i}=x_{i}^{T}\text{β}(\tau )+\epsilon (\tau {)}_{i}\;,\;i=1,\ldots ,n, \end{array}$$

where *τ* is in the range between 0 and 1, $y_{i}$ is the response variable, $x_{i}$ is the vector of predictors, β(τ) is the vector of unknown parameters of interest, and ϵ(τ) is the model error term for the $\tau^{\mathrm{th}}$ quantile. For the sake of notation simplification, we omit *τ* from these parameters.

We wish to estimate the unknown parameters, *β* as $\hat{\text{β}}$ for each $\tau^{\mathrm{th}}$ quantile, which can be done by minimising the check loss function over *β*:

(2) $$\begin{array}{r} \sum_{i=1}^{n}\rho_{\tau }(y_{i}-x_{i}^{T}\text{β}), \end{array}$$

with the check loss function $\rho_{\tau }(\Delta )=\Delta [\tau \cdot I_{\Delta \geq 0}-(1-\tau )\cdot I_{\Delta <0}]$ where $I_{\Delta \geq 0}$ represents the value 1 if Δ belongs to the set [0,∞), and the value 0 otherwise.

Minimising Equation (2) is same as maximising a likelihood function. An asymmetric Laplace distribution (ALD) is employed, which is the common choice for the quantile regression analysis [61,62]. We assume that $\epsilon_{i}∼\mathrm{AL}(0,\sigma ,\tau ),i=1,\ldots ,n$, where the AL(⋅) is the ALD with its density

$$\begin{array}{r} f^{\mathrm{AL}}(\epsilon_{i})=\frac{\tau (1-\tau )}{\sigma }\exp\left\{-\frac{\rho_{\tau }(\epsilon_{i})}{\sigma }\right\}. \end{array}$$

Here, $\rho_{\tau }(\epsilon_{i})$ denotes the usual check loss function of Koenker and Bassett[27].

We are interested in selecting a subset of important predictors, which have adequate explanatory and predictive capabilities. Because regularisation has been shown to be effective in improving the predictive accuracy [34,58], one of the common procedures for simultaneously facilitating the parameter estimation and variable selection is to impose a penalty function on the likelihood to arrive at the penalised loss function,

(3) $$\begin{array}{r} \sum_{i=1}^{n}\rho_{\tau }(y_{i}-x_{i}^{T}\text{β})+P(\text{β},\delta ), \end{array}$$

which is minimised to obtain the $\tau^{\mathrm{th}}$ regularised quantile regression estimator. Here, P(β,δ) is a regularisation penalty function and *δ* is a penalty parameter that controls the level of sparsity. Typically, Bayesian regularised quantile regression is formulated through the relationship between the penalised loss function and the ALD.

Bayesian inference is one of the most popular approaches for the regression analysis. It makes inference for an entire posterior distribution of a parameter of interest, as well as incorporation of parameter uncertainty and prior information about data. This encourages the use of Bayesian analysis over standard frequentist approaches.

By using the identity of Andrews and Mallows [5],

$$\begin{array}{r} \exp(-|\mathrm{ab}|)=\int_{0}^{\infty }\frac{a}{\sqrt{2\pi \nu }}\exp\left\{-\frac{1}{2}(a^{2}\nu +b^{2}\nu^{-1})\right\}d\nu , \end{array}$$

for any *a*,*b*>0, letting $a=1/\sqrt{2\sigma }$ & $b=\epsilon /\sqrt{2\sigma }$ and multiplying a factor of exp⁡(−(2τ−1)ϵ/2σ), to express the probability density function of the ALD errors as its normal scale mixture representation,

$$\begin{array}{r} f_{\mathrm{AL}}(\epsilon_{i})=\int_{0}^{\infty }\frac{1}{\sqrt{4\pi \sigma^{3}v_{i}}}\exp\left\{-\frac{(\epsilon_{i}-(1-2\tau )v_{i}{)}^{2}}{4\sigma v_{i}}-\frac{\tau (1-\tau )v_{i}}{\sigma }\right\}dv_{i}\;, \end{array}$$

as proposed by Reed and Yu [42] and Hideo and Kobayashi [24]. This representation can be utilised to facilitate Gibbs sampling algorithms [14,22,24,29].

Rather than the standard linear model, we will use the FP model to develop the non-linear model under Bayesian quantile regression and variable selection.

### 2.2. Fractional polynomials

Box and Tidwell [9] introduced the transformation now known as the Box-Tidwell transformation,

$$\begin{array}{r} x^{\left(a\right)}=\left\{ \begin{array}{ll} x^{a}, & \mathrm{if}\;a\neq 0\;,\\ \log(x), & \mathrm{if}\;a=0\;, \end{array}\right. \end{array}$$

where *a* is a real number. Royston and Altman [43] extended the classical polynomials to a class which they called FPs.

An FP of degree *m* with powers $p_{1}\leq \ldots \leq p_{m}$ and corresponding coefficients $\alpha_{1},\ldots ,\alpha_{m}$ is

$$\begin{array}{r} f^{m}(x;\alpha ,p)=\sum_{j=1}^{m}\alpha_{j}h_{j}(x)\;, \end{array}$$

where $h_{0}(x)=1$ and

(4) $$\begin{array}{r} h_{j}(x)=\left\{ \begin{array}{ll} x^{\left(p_{j}\right)}, & \mathrm{if}p_{j}\neq p_{j-1},\\ h_{j-1}(x)\log(x), & \mathrm{if}p_{j}=p_{j-1}, \end{array}\right. \end{array}$$

where j=1…,m. Note that the definition $h_{j}(x)$ allows the repeated powers. The bracket around the exponent denote the Box-Tidwell transformation (Equation (4)). For m≤3, Royston and Altman [44] constrained the set of possible powers $p_{j}$ to the set

(5) $$\begin{array}{r} S=\left\{-2,-1,-\frac{1}{2},0,\frac{1}{2},1,2,3\right\}, \end{array}$$

which encompasses the classical polynomial powers 1, 2, 3, yet also offers square roots and reciprocals. Royston and Sauerbrei [45] argued that this set is sufficient to approximate all powers in internals [−2,3]. The simple example of the FP model is as follows. An FP with *m*=3 powers and its power vector $p=(p_{1},p_{2},p_{3})=(-1/2,2,2)$ would be

$$\begin{array}{r} f^{3}(x;\alpha ,p)=\alpha_{1}x^{-1/2}+\alpha_{2}x^{2}+\alpha_{3}x^{2}\log(x), \end{array}$$

where the last term reflects the repeated power 2.

Generalisation to the case of multiple predictors:

(6) $$\begin{array}{r} \eta (x)=\sum_{l=1}^{k}f_{l}^{m_{l}}(x_{l};\alpha_{l},p_{l})=\sum_{l=1}^{k}\sum_{j=1}^{m_{l}}\alpha_{\mathrm{lj}}h_{\mathrm{lj}}(x_{l})\;. \end{array}$$

This is called the multiple FP model. Suppose we continue examining *k* continuous predictors $x_{1},\ldots ,x_{k}$ and content themselves with a maximum degree of $m_{\max}\leq 3$ for each $f_{l}^{m_{l}}$, for instance, $0\leq m_{l}\leq m_{\max}$ for l=1,…,k, where $m_{l}=0$ denotes the omission of $x_{l}$ from the model. From the powers set S, $m_{l}$ powers are chosen, which need not be different due to the inclusion of logarithmic terms for repeated powers (Equation (4)), we now employ the $\tau^{\mathrm{th}}$ non-linear quantile regression with the normal scale mixture representation of the ALD errors,

(7) $$\begin{array}{r} y=B\text{β}+\theta_{1}v+\sqrt{\theta_{2}v\sigma^{2}}z\;, \end{array}$$

where the (n×D)-matrix B is a function of the $l^{\mathrm{th}}$ predictor for the $i^{\mathrm{th}}$ observations, $x_{l}$ ( i=1,…,n, and l=1,…,k), the unknown parameter vector $\text{β}=(\alpha_{1},\ldots ,\alpha_{k}{)}^{T}$ with $\alpha_{l}=(\alpha_{l1},\ldots ,\alpha_{lm_{l}})$ for l=1,…,k, $v=(v_{1},\ldots ,v_{n}{)}^{T}$ is a vector of exponential random variables with a rate of τ(1−τ)/σ, $z=(z_{1},\ldots ,z_{n}{)}^{T}$ is a vector of standard normal random variables, $z_{i}$ is independent of $v_{i}$ for i=1,…,n, $\theta_{1}=(1-2\tau )/(\tau (1-\tau ))$ and $\theta_{2}=2/(\tau (1-\tau ))$. Each entry of matrix B is a vector, $B_{\mathrm{id}}=B(x_{\mathrm{id}})=(h_{l1}(x_{\mathrm{il}}),\ldots ,h_{lm_{l}}(x_{\mathrm{il}}){)}^{T}$, for i=1,…,n, l=1,…,k, and d=1,…,D.

A special way of defining the matrix B is through the use of FPs. In this case, the basis function $B(x_{l})$ is chosen as the transformation $h_{\mathrm{lj}}$ in Equation (6) ( $j=1,\ldots ,m_{l}$). The transformation $h_{j}$ is determined by the power vector $p_{1},\ldots ,p_{k}$ through their definition (Equation (4)). Note that the $p_{l}$ is empty if the predictor $x_{l}$ is not included in the model ( $m_{l}=0$).

### 2.3. Bayesian approach and variable selection

Given the model in Equation (7), the likelihood function conditional on $\text{β}=({\text{β}}_{1},\ldots ,{\text{β}}_{D}{)}^{T}$, *σ*, $v=(v_{1},\ldots ,v_{n}{)}^{T}$ can be written as

$$\begin{array}{r} f(y|\text{β},\sigma ,v,B)=\prod_{i=1}^{n}\frac{1}{\sqrt{4\pi \sigma^{3}v_{i}}}\exp\left\{-\frac{(y_{i}-B(x_{i}{)}^{T}\text{β}-(1-2\tau )v_{i}{)}^{2}}{4\sigma v_{i}}-\frac{\tau (1-\tau )v_{i}}{\sigma }\right\}\;. \end{array}$$

We employ the three-stage algorithm of Dao et al. [17] for Bayesian non-linear quantile regression with variable selection. It can be summarised, as follows.

The first-stage is the expectation-maximisation (EM) algorithm consisting of two main steps: the Expectation step (E step) and the Maximum step (M step). Dempster et al. [18] proposed the EM algorithm, which is a statistical simulation method and it aims to solve the complex data analysis problem with missing data.

Suppose the complete data (y,v) is composed of the observed data $y=(y_{1},\ldots ,y_{n}{)}^{T}$ and missing data $v=(v_{1},\ldots ,v_{n}{)}^{T}$, whereas $B(x_{i})$, i=1,…,n, is treated as a function of fixed predictors. Maximum likelihood estimates can be obtained by maximising log-likelihood function log⁡f(β,σ|y,v) of the complete data. The EM algorithm has the following two steps: the E step and the M step.

- [E step] Given initial values of ${\text{β}}^{\left(0\right)}$ and $\sigma^{\left(0\right)}$, we denote ${\text{β}}^{\left(q-1\right)}$ and $\sigma^{\left(q-1\right)}$ as the $(q-1{)}^{\mathrm{th}}$ iteration value of parameters β and *σ* in the EM algorithm, and we define the mathematical expectation of the complete data as a Q-function
$$\begin{array}{r} Q(\text{β},\sigma |y,{\text{β}}^{\left(q-1\right)},\sigma^{\left(q-1\right)})=E_{y,{\text{β}}^{\left(q-1\right)},\sigma^{\left(q-1\right)}}[\log f(\text{β},\sigma |y,v)]\;. \end{array}$$
- [M step] We obtain the updated values of ${\text{β}}^{\left(q\right)}$ and $\sigma^{\left(q\right)}$ by maximising $Q(\text{β},\sigma |y,{\text{β}}^{\left(q-1\right)},\sigma^{\left(q-1\right)})$ over parameters β and *σ*:
$$\begin{array}{r} {\text{β}}^{\left(q\right)}=(B^{T}W^{\left(q-1\right)}B{)}^{-1}B^{T}W^{\left(q-1\right)}(y-\theta_{1}\Delta 3)\;, \end{array}$$
  where
$$\begin{array}{rl} & \Delta 3={\left(\left|y_{1}-B(x_{1}{)}^{T}{\text{β}}^{\left(q-1\right)}\right|,\ldots ,{\left|y_{n}-B(x_{n}{)}^{T}\text{β}\right|}^{\left(q-1\right)}\right)}^{T}\;,\\ & W^{\left(q-1\right)}=\mathrm{diag}(1/\Delta 3_{1},\ldots ,1/\Delta 3_{n})\;, \end{array}$$
  and
$$\begin{array}{r} \sigma^{\left(q\right)}=\frac{1}{2(3n+2)}\;\left\{\sum_{i=1}^{n}\Delta 2_{i}+\sum_{i=1}^{n}\frac{(y_{i}-B(x_{i}{)}^{T}{\text{β}}^{\left(q\right)}{)}^{2}}{\Delta 3_{i}}-2\theta_{1}\sum_{i=1}^{n}(y_{i}-B(x_{i}{)}^{T}{\text{β}}^{\left(q\right)})\;\right\}\;, \end{array}$$
  where $\Delta 2_{i}=|y_{i}-B(x_{i}{)}^{T}{\text{β}}^{\left(q-1\right)}|+2\sigma^{\left(q-1\right)}\mathrm{for}i=1,\ldots ,n$.

Repeat both E-step and M-step until the EM algorithm meets the required condition, then the final iteration values are set as the posterior modes of β and *σ*, denoted by $\tilde{\text{β}}$ and $\tilde{\sigma }$, respectively.

The second-stage algorithm is the Gibbs sampling algorithm. The quantile-specific Zellner's *g*-prior [3] is used for the prior specification and it is given by

(8) $$\begin{array}{r} \text{β}|\sigma ,V,B∼N\left(0,2\mathrm{\sigma g}\Sigma_{v}^{-1}\right)\;\mathrm{and}\;p(\sigma )\propto \frac{1}{\sigma }\;, \end{array}$$

where N(⋅) is the multivariate normal distribution, *g* is a scaling factor, $V=\mathrm{diag}(1/v_{1},\ldots ,1/v_{n})$, and $\Sigma_{v}=B^{T}\mathrm{VB}$. This prior specification has an advantage, as it contains information that is dependent upon the quantile levels, which increases posterior inference accuracy.

Given the posterior modes, $\tilde{\text{β}}$ and $\tilde{\sigma }$ as the starting value, we denote ${\text{β}}^{\left(r-1\right)}$ and $\sigma^{\left(r-1\right)}$ as the $(r-1{)}^{\mathrm{th}}$ iteration value of parameters β and *σ* in the Gibbs sampling algorithm.

- Sample $v_{i}^{\left(r\right)}$ from
$$\begin{array}{r} p(v_{i}|y,{\text{β}}^{\left(r-1\right)},\sigma^{\left(r-1\right)})∼\mathrm{GIG}\left(0,\frac{1}{2\sigma },\frac{(y_{i}-B(x_{i}{)}^{T}\text{β}{)}^{2}+\frac{1}{g}{\text{β}}^{T}B(x_{i})B(x_{i}{)}^{T}\text{β}}{2\sigma }\right), \end{array}$$
  for i=1,…,n, and GIG(0,c,d) is the generalised inverse Gaussian with its density
$$\begin{array}{r} f^{\mathrm{GIG}}(v)=\frac{1}{2K_{0}(\sqrt{\mathrm{cd}})}v^{-1}\exp\left(-\frac{1}{2}(\mathrm{cv}+dv^{-1})\right),\;v>0\;, \end{array}$$
  where $K_{0}(\cdot )$ is the modified Bessel function of the second kind at index 0 [6].
- Sample $\sigma^{\left(r\right)}$ from
$$\begin{array}{r} p(\sigma |y,v^{\left(r\right)})∼\mathrm{IG}\left(\frac{3n}{2},\frac{1}{4}(y-\theta_{1}v{)}^{T}VH_{v}(y-\theta_{1}v)+\frac{2}{\theta_{2}}\sum_{i=1}^{n}v_{i}\right), \end{array}$$
  where IG(⋅) is the inverse Gamma distribution, $H_{v}=I_{n}-g/(g+1)B\Sigma_{v}^{-1}B^{T}V$.
- Sample ${\text{β}}^{\left(r\right)}$ from
$$\begin{array}{r} p(\text{β}|y,v^{\left(r\right)},\sigma^{\left(r\right)})∼N\left(\frac{g}{g+1}\Sigma_{v}^{-1}B^{T}V(y-\theta_{1}v),\frac{2\mathrm{\sigma g}}{g+1}\Sigma_{v}^{-1}\right)\;. \end{array}$$
- Calculate the important weights
$$\begin{array}{r} w^{\left(r\right)}=\frac{p({\text{β}}^{\left(r\right)},\sigma^{\left(r\right)},v^{\left(r\right)}|y)}{p({\text{β}}^{\left(r\right)}|v^{\left(r\right)},\sigma^{\left(r\right)},y)p(\sigma^{\left(r\right)}|v^{\left(r\right)},y)p(v^{\left(r\right)})}\;, \end{array}$$
  based on $v^{\left(r\right)}$, $\sigma^{\left(r\right)}$ and ${\text{β}}^{\left(r\right)}$. This is to adjust for the GIG approximation of the marginal posterior of v given y, which is given by its unnormalised density function
$$\begin{array}{r} \pi (v|y)\propto \frac{p(v|\tilde{\text{β}},\tilde{\sigma },y)}{p(\tilde{\text{β}}|y,v.\tilde{\sigma })p(\tilde{\sigma }|y,v)}\;, \end{array}$$
  where $p(v|\tilde{\text{β}},\tilde{\sigma },y)$ is an importance sampling density in the importance sampling algorithm. The importance weights will be used to determine the acceptance probability of each $\left\{{\text{β}}^{\left(r\right)},\sigma^{\left(r\right)},v^{\left(r\right)}\right\}$.

The algorithm iterates until the Gibbs sampling algorithm reaches the final MCMC iteration indexed at R and discard the burn-in period.

Finally, the third-stage is the important re-weighting step. The *S* samples are drawn from the importance weights without replacement where *S*<*R* is the number of importance weighting steps. A random indicator vector $\gamma =(\gamma_{1},\ldots ,\gamma_{D}{)}^{T}$ is introduced to the non-linear model

$$\begin{array}{r} M_{\gamma }:y=B_{\gamma }\text{β}+\epsilon \;, \end{array}$$

where $B_{\gamma }$ is the $\left(n\times D_{\gamma }\right)$ matrix consisting of important predictors and ${\text{β}}_{\gamma }$ of length $D_{\gamma }$ is the non-zero parameter vector. The same prior specification in Equation (8) is employed along with a prior on $\gamma_{d}$, d=1,…,D, and a beta prior on *π*:

$$\begin{array}{r} p(\gamma |\pi )\propto \pi^{\sum_{d=1}^{D}\gamma_{d}}(1-\pi {)}^{D-\sum_{d=1}^{D}\gamma_{d}}\;\mathrm{and}\;p(\pi )∼\mathrm{Beta}\left(\frac{1}{2},\frac{1}{2}\right)\;, \end{array}$$

where π∈[0,1] is the prior probability of randomly including a predictor in the model. Then, *π* is marginalised out from p(γ|π) resulting as

$$\begin{array}{r} p(\gamma )\propto \mathrm{Beta}\left(\sum_{d=1}^{D}\gamma_{d}+\frac{1}{2},D-\sum_{d=1}^{D}\gamma_{d}+\frac{1}{2}\right)\;. \end{array}$$

The marginal likelihood of y under the model $M_{\gamma }$ is then obtained by integrating out β and *σ* resulting as

$$\begin{array}{r} p(y|\gamma ,v)∼t_{2n}\left((1-2\tau )v,\frac{4\sum_{i=1}^{n}v_{i}}{\sigma \theta_{2}}{\left(V-\frac{g}{g+1}VB_{\gamma }\Sigma_{v}(\gamma {)}^{-1}B_{\gamma }^{T}V\right)}^{-1}\right)\;, \end{array}$$

where $t_{2n}(\cdot )$ is the multivariate Student t-distribution with 2n degrees of freedom. The posterior probability of $M_{\gamma }$ is therefore given by p(γ|y,v)∝p(y|γ,v)p(γ). Lastly, the independent samples of v from the second-stage algorithm are drawn based on the *S* samples and the important re-weighting step is iterated until the *S* samples of γ are obtained. Then, the posterior inclusion probability is estimated, as follows

$$\begin{array}{r} \hat{p}(\gamma_{d}=1|y,v)=\frac{1}{\tilde{S}}\sum_{s=1}^{\tilde{S}}\gamma_{d}^{\left(s\right)}\;,\;d=1,\ldots ,D\;, \end{array}$$

where $\tilde{S}$ is the number of iterations after discarding the burn-in period.

## 3. Data preparation and data analysis

This study is based on the data of the NHANES during 2007–2008. The survey conducted by the National Center for Health Statistics of the Centers for Disease Control and Prevention used a complex, stratified, multistage sampling design to select a representative sample of non-institutionalised population in US civilians to participate in a series of comprehensive health-related interviews and examinations. In total, 12,943 people participated in the NHANES 2007–2008 study.

The study variables included SBP and DBP as the response variables. The BP measurements were taken as follows. After a resting period of 5 minutes in a sitting position and determination of maximal inflation level, three consecutive BP readings were recorded. A fourth reading was recorded if a BP measurement is interrupted or incomplete. All the results were taken in the Mobile Examination Center. The BP measurements are essential for hypertension screening and disease management, since hypertension is an important risk factor for cardiovascular and renal disease. Then, in this study, SBP and DBP were selected as response variables where each was averaged over the second and third readings. Predictor variables were BMI, age, ethnicity, gender and marital status.

We initially included 9 762 participants who have completed both BP and body measure examinations in the study. From 9 762 participants, we excluded those who had not underwent examinations. Then, amongst the remaining 4 612 participants, we further excluded those who refused to reveal their marital status. Finally, 4 609 participants were included for analysis in this study.

The NHANES protocols were approved by the National Center for Health Statistics research ethics review boards, and informed consent was obtained from all participants. The research adhered to the tenets of the Declaration of Helsinki.

The R version 4.2.2 was used to conduct both frequentist and Bayesian analyses. Both 'quantreg' and 'Brq' R packages were employed to fit the frequentist and Bayesian approaches of the quantile regression model with FPs, respectively. The source R code was provided from the main author of Dao et al. [17] to fit the Bayesian quantile regression with variable selection and FPs via the three-stage algorithm.

This study considered two quantile models at the $50^{\mathrm{th}}$, $75^{\mathrm{th}}$ and $95^{\mathrm{th}}$ percentiles. When modelling hypertension, it is preferable to model both median and extremely high values of SBP and DBP, which correspond to the median and upper distributions of SBP and DBP, respectively [31]. The following two quantile models were used for the analysis for the fixed quantile level *τ*:

$$\begin{array}{rl} {\mathrm{SBP}}_{i} & ={\mathrm{BMI}}_{i}{\text{β}}_{1}+{\mathrm{BMI}}_{i}^{0.5}{\text{β}}_{2}+{\mathrm{Age}}_{i}{\text{β}}_{3}+{\mathrm{Age}}_{i}^{0.5}{\text{β}}_{4}+{\mathrm{Ethnicity}}_{i}{\text{β}}_{5}+{\mathrm{Gender}}_{i}{\text{β}}_{6}\\ & \;+{\mathrm{MaritalStatus}}_{i}{\text{β}}_{7}\;,\\ {\mathrm{DBP}}_{i} & ={\mathrm{BMI}}_{i}{\text{β}}_{1}+{\mathrm{BMI}}_{i}^{0.5}{\text{β}}_{2}+{\mathrm{Age}}_{i}{\text{β}}_{3}+{\mathrm{Age}}_{i}^{0.5}{\text{β}}_{4}+{\mathrm{Ethnicity}}_{i}{\text{β}}_{5}+{\mathrm{Gender}}_{i}{\text{β}}_{6}\\ & \;+{\mathrm{MaritalStatus}}_{i}{\text{β}}_{7}\;, \end{array}$$

for i=1,…,4609.

The power of 0.5 was chosen for continuous variables, including BMI and age. The remaining variables were linear because they are categorical. Similar FP models were employed to model BP within the linear regression framework, see [19,49,51] and amongst others.

## 4. Results

In this section, both descriptive and model analyses are provided for the NHANES 2007–2008 dataset using the proposed model. To evaluate the performance of the proposed model, we included two existing methods, including quantile regression and Bayesian quantile regression, with the FP model for a fair comparative analysis. The model comparison is discussed outlining the advantages of the proposed model over these two methods. All the results are provided in this section through tables and figures for each regression analysis.

### 4.1. Descriptive analysis

For this analysis, continuous variables were collapsed into categorical variables, including SBP, DBP, BMI and age. According to the guidelines of Whelton et al. [54], the BP variables were divided into three groups: normal ( <120 mmHg for SBP, <80 mmHg for DBP), pre-hypertension (120−139 mmHg for SBP, 80−89 mmHg for DBP) and hypertension ( ≥140 mmHg for SBP, ≥90 mmHg for DBP). The BMI variable was also divided into six groups: underweight ( <18.5), healthy (18.5−24.9), overweight (25−29.9), obese (30−34.9), very obese (35−39.9) and morbidly obese ( ≥40) [13].

Tables 1 and 2 report SBP and DBP proportions amongst US adults by demographic and lifestyle characteristics, including BMI, age, ethnicity, gender and marital status. The Cramér's V value was used to measure the magnitude of the association between SBP, DBP, socio-demographic characteristics and BMI of the participants. Their values with p-values are also presented in Tables 1-2 and compared with with guidelines given by Rea and Parker (2014) [41]: 0.00 to under 0.10 = very weak association, 0.10 to under 0.20 = weak association, 0.20 to under 0.40 = moderate association and 0.40 and above = strong association.

**Table 1.** SBP amongst US adults by BMI and socio-demographic characteristics.

		Normal BP (<120 mmHg)	Pre-Hypertension (120–139 mmHg)	Hypertension (≥140 mmHg)
BMI	Underweight	37 (56.92%)	16 (24.62%)	12 (18.46%)
	Healthy	734 (60.31%)	343 (28.18%)	140 (11.50%)
	Overweight	781 (49.49%)	565 (35.80%)	232 (14.70%)
	Obese	415 (41.71%)	414 (41.61%)	166 (16.68%)
	Very obese	201 (42.68%)	187 (39.70%)	83 (17.62%)
	Morbidly obese	106 (37.46%)	116 (40.99%)	61 (21.55%)
P-value (Cramér's V value)		P-value < 0.01 (0.1106)		
Age	20–29 years	493 (73.36%)	164 (24.40%)	15 (2.23%)
	30–39 years	543 (65.66%)	251 (30.35%)	33 (3.99%)
	40–49 years	460 (55.89%)	285 (34.63%)	78 (9.48%)
	≥50 years	778 (34.02%)	941 (41.15%)	568 (24.84%)
		P-value < 0.01 (0.2535)		
Ethnicity	Mexican American	456 (54.29%)	279 (33.21%)	105 (12.50%)
	Other Hispanic	286 (53.16%)	186 (34.57%)	66 (12.27%)
	Non-Hispanic white	1006 (47.61%)	793 (37.53%)	314 (14.86%)
	Non-Hispanic black	425 (45.31%)	324 (34.54%)	189 (20.15%)
	Other non-Hispanic race	101 (56.11%)	59 (32.78%)	20 (11.11%)
		P-value < 0.01 (0.0665)		
Gender	Male	999 (43.28%)	957 (41.46%)	352 (15.25%)
	Female	1275 (55.41%)	684 (29.73%)	342 (14.86%)
		P-value < 0.01 (0.1310)		
Marital	Married	1219 (48.39%)	927 (36.80%)	373 (14.81%)
Status	Widowed	84 (30.11%)	103 (36.92%)	92 (32.97%)
	Divorced	226 (44.14%)	182 (35.55%)	104 (20.31%)
	Separated	89 (52.05%)	57 (33.33%)	25 (14.62%)
	Never married	468 (58.87%)	256 (32.20%)	71 (8.93%)
	Living with partner	188 (56.46%)	116 (34.83%)	29 (8.71%)
		P-value < 0.01 (0.1251)

**Table 2.** DBP amongst US adults by BMI and socio-demographic characteristics.

		Normal BP (<80 mmHg)	Pre-Hypertension (80-89 mmHg)	Hypertension (≥90 mmHg)
BMI	Underweight	49 (75.38%)	12 (18.46%)	4 (6.15%)
	Healthy	1025 (84.22%)	148 (12.16%)	44 (3.62%)
	Overweight	1265 (80.16%)	243 (15.40%)	70 (4.44%)
	Obese	772 (77.59%)	168 (16.88%)	55 (5.53%)
	Very obese	356 (75.58%)	78 (16.56%)	37 (7.86%)
	Morbidly obese	217 (76.68%)	47 (16.61%)	19 (6.71%)
P-value (Cramér's V value)		P-value < 0.01 (0.0587)		
Age	20–29 years	619 (92.11%)	47 (6.99%)	6 (0.89%)
	30–39 years	681 (82.35%)	118 (14.27%)	28 (3.39%)
	40–49 years	584 (70.96%)	173 (21.02%)	66 (8.02%)
	≥50 years	1800 (78.71%)	358 (15.65%)	129 (5.64%)
		P-value < 0.01 (0.1118)		
Ethnicity	Mexican American	699 (83.21%)	116 (13.81%)	25 (2.98%)
	Other Hispanic	444 (82.53%)	70 (13.01%)	24 (4.46%)
	Non-Hispanic white	1687 (79.84%)	327 (15.48%)	99 (4.69%)
	Non-Hispanic black	711 (75.80%)	154 (16.42%)	73 (7.78%)
	Other non-Hispanic race	143 (79.44%)	29 (16.11%)	8 (4.44%)
		P-value < 0.01 (0.0569)		
Gender	Male	1732 (75.04%)	423 (18.33%)	153 (6.63%)
	Female	1952 (84.83%)	273 (11.86%)	76 (3.30%)
		P-value < 0.01 (0.1244)		
Marital	Married	2017 (80.07%)	385 (15.28%)	117 (4.64%)
Status	Widowed	231 (82.80%)	38 (13.62%)	10 (3.58%)
	Divorced	386 (75.39%)	87 (16.99%)	39 (7.62%)
	Separated	133 (77.78%)	26 (15.20%)	12 (7.02%)
	Never married	656 (82.52%)	103 (12.96%)	36 (4.53%)
	Living with partner	261 (78.38%)	57 (17.12%)	15 (4.50%)
		P-value = 0.0516 (0.0444)		

It is evident from Tables 1 and 2 that hypertension was more prevalent in underweight, very obese and morbidly obese participants for both BP measures where the very obese and morbidly obese had the highest prevalence for DBP and SBP measures, respectively. The same trend is observed on the proportions of elevated BP for DBP measure. It is clear that healthy participants had the highest prevalence of normal BP for both BP measures.

Concerning age, the prevalence of both elevated BP and hypertension increased with age, with the 40–49 years age group having the highest proportions for DBP measure and the 50 years and above age group for SBP measure. In regards to ethnicity, the non-Hispanic Black participants had the highest prevalence of hypertension compared to other races for both BP measures.

Tables 1 and 2 also show that men had the highest prevalence of both elevated BP and hypertension for both BP measures. Participants who were separated or divorced and those who became widowed had the highest prevalence of hypertension for DBP and SBP measures, respectively.

Lastly, at the 1% significance level, Tables 1 and 2 exhibit very weak to weak associations between BP measures, BMI and socio-demographic characteristics amongst US adults. However, there is a moderate association between SBP measure and age. There is no statistically significant association between DBP measure and marital status at the 5% level.

### 4.2. Model analysis

Tables 3 and 4 provide the coefficients for predictors relating to SBP and DBP responses for three quantile regression models with FPs at three quantile levels ( τ=0.50,0.75,0.95), including one frequentist and two Bayesian approaches with one using variable selection. For Bayesian approaches, parameters were obtained via posterior men. The 95% confidence intervals were also obtained for the frequentist approach, whilst the 95% credible intervals were obtained for the Bayesian approaches. A confidence interval describes a probability, for instance, if a user constructs a confidence interval with some confidence level then they are confident that an estimate would fall within the interval. On the other hand, a credible interval is an interval in the domain of a posterior probability distribution where an unobserved parameter value falls with a particular probability. We denote the frequentist approach as the QR-FP model, and two Bayesian approaches as the BQR-FP and BQRVS-FP models where the latter uses variable selection.

**Table 3.** One frequentist and two Bayesian quantile regression analyses for relationship between SBP and risk factors.

Quantile Regression				
	*τ*	0.50	0.75	0.95
BMI		−2.856(−3.278,−2.280)	−2.198(−3.040,−1.715)	−2.024(−3.141,−0.798)
${\text{BMI}}^{0.5}$		36.085 (29.932, 40.529)	29.210 (23.907, 38.130)	29.113 (15.239, 42.302)
Age		0.510 (0.130, 0.785)	0.317(−0.003,0.885)	0.710(−0.220,1.630)
${\text{Age}}^{0.5}$		−1.758(−5.430,3.339)	3.297(−4.116,7.654)	2.300(−9.906,14.672)
Ethnicity		0.626 (0.154, 1.040)	0.995 (0.366, 1.495)	1.214 (0.199, 2.642)
Gender		−4.323(−5.302,−3.512)	−3.813(−5.231,−2.506)	−3.278(−6.147,−0.762)
Marital Status		0.894 (0.612, 1.155)	1.327 (0.916, 1.746)	1.400 (0.650, 2.037)
Bayesian Quantile				
Regression				
	*τ*	0.50	0.75	0.95
BMI		−2.818(−3.208,−2.447)	−2.255(−2.669,−1.889)	−2.120(−2.603,−1.685)
${\text{BMI}}^{0.5}$		35.628 (31.653, 39.794)	29.825 (25.763, 34.419)	30.191 (25.146, 35.809)
Age		0.484 (0.233, 0.734)	0.364 (0.103, 0.664)	0.768 (0.428, 1.142)
${\text{Age}}^{0.5}$		−1.366(−4.737,2.002)	2.735(−1.237,6.249)	1.446(−3.550,6.077)
Ethnicity		0.640 (0.288, 0.979)	0.957 (0.561, 1.359)	1.341 (0.839, 1.829)
Gender		−4.376(−5.138,−3.645)	−3.809(−4.784,−2.823)	−3.346(−4.397,−2.190)
Marital Status		0.888 (0.656, 1.125)	1.347 (1.055, 1.637)	1.354 (1.041, 1.649)
Bayesian Quantile				
Regression Fractional				
Polynomials &				
Variable Selection				
	*τ*	0.50	0.75	0.95
BMI		−2.812(−3.164,−2.468)	−2.581(−2.974,−2.168)	−2.426(−2.813,−2.027)
${\text{BMI}}^{0.5}$		35.547 (31.789, 39.269)	33.335 (28.817, 37.747)	33.335 (28.815, 37.784)
Age		0.459 (0.226, 0.680)	0.537 (0.274, 0.806)	0.945 (0.643, 1.256)
${\text{Age}}^{0.5}$		−1.129(−4.197,2.029)	−0.051(−3.717,3.536)	−1.382(−5.473,2.680)
Ethnicity		0.571 (0.258, 0.898)	0.843 (0.484, 1.212)	1.152 (0.753, 1.616)
Gender		−4.577(−5.300,−3.899)	−4.291(−5.053,−3.518)	−4.343(−5.301,−3.351)
Marital Status		0.828 (0.632, 1.033)	1.139 (0.893, 1.381)	1.331 (1.052, 1.617)

**Table 4.** One frequentist and two Bayesian quantile regression analyses for relationship between DBP and risk factors.

Quantile Regression				
	*τ*	0.50	0.75	0.95
BMI		1.174 (0.705, 1.496)	0.761 (0.507, 1.096)	0.582 (0.022, 1.572)
${\text{BMI}}^{0.5}$		−12.200(−15.675,−7.071)	−7.179(−10.821,−4.242)	−3.995(−13.869,2.247)
Age		−2.266(−2.477,−1.979)	−2.018(−2.252,−1.832)	−1.852(−2.418,−1.418)
${\text{Age}}^{0.5}$		31.329 (27.308, 34.170)	28.298 (25.758, 31.451)	26.918 (21.199, 34.557)
Ethnicity		0.561 (0.203, 0.841)	0.712 (0.411, 1.030)	1.264 (0.345, 2.013)
Gender		−3.345(−4.160,−2.651)	−3.619(−4.337,−2.976)	−4.592(−5.769,−3.047)
Marital Status		0.210(−0.041,0.448)	0.368 (0.171, 0.549)	0.466 (0.143, 0.934)
Bayesian Quantile				
Regression				
	*τ*	0.50	0.75	0.95
BMI		1.153 (0.836, 1.433)	0.798 (0.539, 1.056)	0.656 (0.345, 0.974)
${\text{BMI}}^{0.5}$		−11.923(−15.007,−8.505)	−7.554(−10.406,−4.748)	−4.624(−7.981,−1.332)
Age		−2.253(−2.431,−2.058)	−2.040(−2.224,−1.863)	−1.870(−2.064,−1.663)
${\text{Age}}^{0.5}$		31.131 (28.434, 33.566)	28.594 (26.243, 31.077)	27.176 (24.467, 29.773)
Ethnicity		0.536 (0.291, 0.777)	0.706 (0.455, 0.966)	1.328 (0.981, 1.667)
Gender		−3.391(−3.999,−2.778)	−3.635(−4.169,−3.109)	−4.498(−5.086,−3.924)
Marital Status		0.220 (0.030, 0.408)	0.374 (0.222, 0.533)	0.484 (0.304, 0.667)
Bayesian Quantile				
Regression Fractional				
Polynomials &				
Variable Selection				
	*τ*	0.50	0.75	0.95
BMI		1.101 (0.823, 1.381)	0.808 (0.568, 1.041)	0.874 (0.584, 1.147)
${\text{BMI}}^{0.5}$		−11.299(−14.374,−8.289)	−7.620(−10.207,−4.940)	−7.217(−10.158,−4.080)
Age		−2.217(−2.397,−2.033)	−2.031(−2.203,−1.867)	−2.018(−2.206,−1.821)
${\text{Age}}^{0.5}$		30.603 (28.089, 33.030)	28.381 (26.127, 30.639)	29.063 (26.415, 31.577)
Ethnicity		0.505 (0.278, 0.727)	0.630 (0.391, 0.868)	1.043 (0.747, 1.319)
Gender		−3.401(−3.934,−2.888)	−3.733(−4.219,−3.233)	−4.436(−5.032,−3.827)
Marital Status		0.193 (0.033, 0.347)	0.371 (0.222, 0.523)	0.454 (0.270, 0.628)

For the BQR-FP model, the algorithm was implemented for 10,000 MCMC iterations and 1 000 MCMC iterations were discarded as a burn-in period. For the BQRVS-FP model, the first-stage algorithm ran for 1 000 EM iterations and repeated for 2 replications. Then, 5 000 MCMC iterations were drawn for the second-stage algorithm, whilst discarding 2 500 MCMC iterations as a burn-in period. Finally, the last algorithm ran for 1 250 important re-weighting steps of which 500 steps were discarded as a burn-in period. The value of *g* was selected as 1 000 for all implementations of the variable selection model.

It is evident from Table 3 that all the risk factors except both linear and non-linear terms of age were found to have statistically significant associations with SBP across the two upper quantile levels according to their 95% confidence intervals containing no zero value under the QR-FP model. Looking at the median level, the linear term had association with SBP under the same approach. When looking at the BQR-FP and BQRVS-FP models, only the non-linear term of age did not have a statistically significant association for all quantile levels. On the other hand, Table 4 observes that all the risk factors including non-linear terms had statistically significant associations with DBP across all quantile levels for all model approaches. Still, when looking at the median level under the QR-FP model, it revealed that the marital status did not have statistically significant association.

Table 3 also observes that the BMI, non-linear term of age and gender have negative associations with SBP, whilst the non-linear term of BMI, age and gender have negative associations with DBP from Table 4 for all three model approaches. Under the SBP model, the coefficients of BMI, ethnicity, gender and marital status increased when the quantile levels increased. The same trend is observed for the coefficients of BMI's non-linear term, age, ethnicity and marital status under the DBP model. Observing the coefficient of age's non-linear term, all models saw the reverse U-shaped trend under the SBP model and on other hand, both QR-FP and BQR-FP models had decreasing trends and the BQRVS-FP had the U-shaped trend under the DBP model. Interestingly, the coefficient of BMI's non-linear term under the SBP model followed the decreasing trend for the QR-FP model, the U-shaped trend for the BQR-FP model and the square-root trend for the BQRVS-FP model.

Convergence of both Bayesian approaches was assessed using the trace plots, the density plots and autocorrelation plots. This is essential to perform various diagnostic tools for assessing the convergence [48]. The convergence diagnostics are useful to check stationarity of the Markov chain or good chain mixing and to verify the accuracy of the posterior estimates [32]. The trace plot is in the form of a time series plot indicating whether it reaches stationarity or not. The density plot represents the stationary distribution of posterior samples approximating the posterior distribution of interest. The autocorrelation plot reports the correlation of posterior samples at each chain step with previous estimates of the same variable, lagged by number of iterations. A decreasing trend indicates that the stationary distribution is more random and less dependent on initial values in the chain [23].

**Figure 1.**
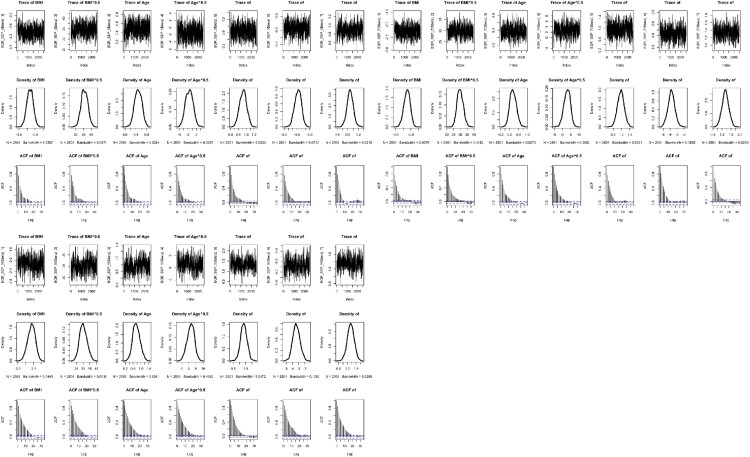
Trace, density and autocorrelation plots for the risk factors of SBP at three quantile levels ( τ=0.5,0.75,0.95) under the Bayesian quantile regression model with FPs.

**Figure 2.**
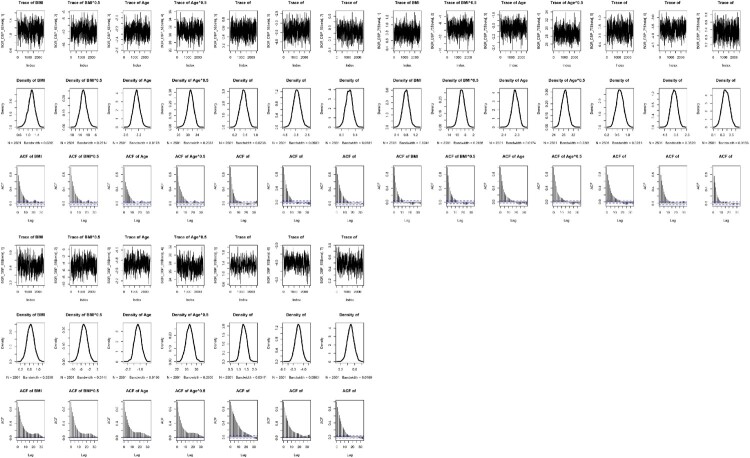
Trace, density and autocorrelation plots for the risk factors of DBP at three quantile levels ( τ=0.5,0.75,0.95) under the Bayesian quantile regression model with FPs.

Figures 1 and 2 present the trace, density and autocorrelation plots for each risk factor of SBP and DBP, respectively under the BQR-FP model. When looking at the trace plots across all the quantile levels, they exhibit stationarity due to relatively constant mean and variance of each plot. Thus, they show the good Markov chain mixing rate. When looking at the density plots across all the quantile levels, they reflect a smooth distribution with one peak at the mode of the distribution indicating a good convergence. It is also shown from the figures that each risk factor of SBP and DBP across all the quantile levels has increasingly random stationary posterior distribution although at the $95^{\mathrm{th}}$ percentile, the trend has a slower decreasing rate.

**Figure 3.**
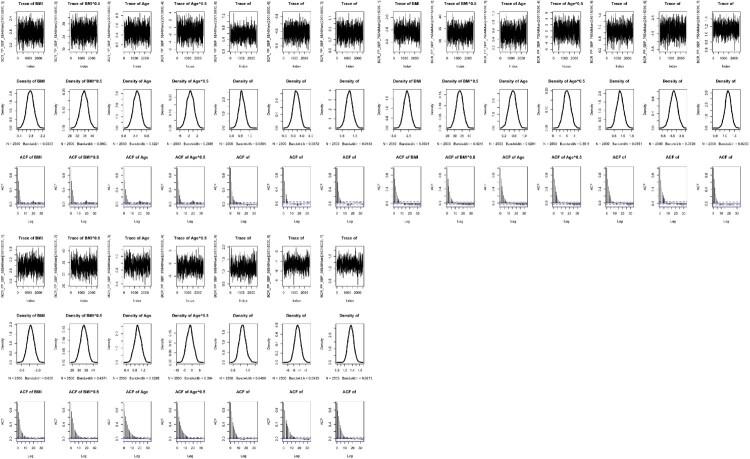
Trace, density and autocorrelation plots for the risk factors of SBP at three quantile levels ( τ=0.5,0.75,0.95) under the Bayesian quantile regression model with FPs and variable selection.

**Figure 4.**
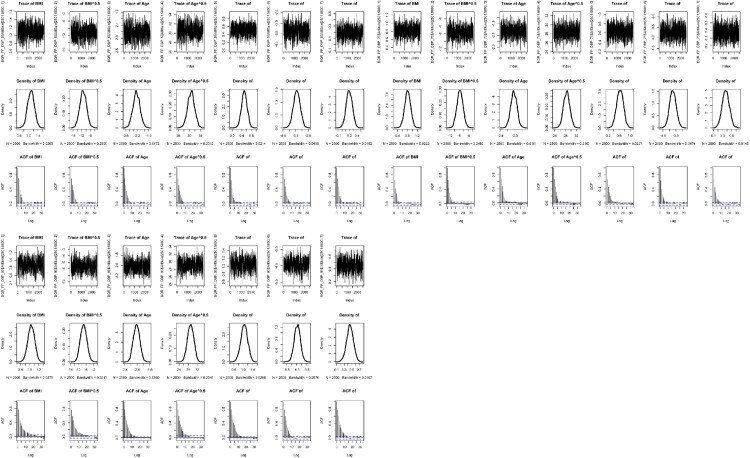
Trace, density and autocorrelation plots for the risk factors of DBP at three quantile levels ( τ=0.5,0.75,0.95) under the Bayesian quantile regression model with FPs and variable selection.

Figures 3 and 4 also present the trace, density and autocorrelation plots for each risk factor of SBP and DBP, respectively under the BQRVS-FP model. All the plots show stationarity, good Markov chain mixing rates and good convergence. Particularly, each autocorrelation plot indicates that their stationary distribution became random and less correlated with the initial values at a faster rate.

Table 5 provides marginal inclusion probabilities (MIPs) that determine which risk factors are influential on SBP and DBP for the BQRVS-FP model at three quantile levels. The risk factors that lie above the threshold of 0.9 of MIP are selected as important predictors. Across all the quantile levels for both SBP and DBP models, all the important risk factors were consistently selected including the non-linear terms. There are two cases of non-important risk factors where, unlike the SBP model, the DBP model did not select marital status at the median level and the SBP model did not select the non-linear term of age at all the quantile levels. This mostly agreed with findings on 95% credible intervals from Tables 3 and 4.

**Table 5.** Selected predictors for both SBP and DBP models via the BQRVS-FP approach at different quantile levels ( τ=0.50,0.75,0.95).

	Model	BMI	${\mathrm{BMI}}^{0.5}$	Age	${\mathrm{Age}}^{0.5}$	Ethnicity	Gender	MaritalStatus
τ=0.50	SBP	1.0000	1.0000	0.9920	0.3062	0.9733	1.0000	1.0000
	DBP	1.0000	1.0000	1.0000	1.0000	0.9973	1.0000	0.8628
τ=0.75	SBP	1.0000	1.0000	0.9920	0.2423	1.0000	1.0000	1.0000
	DBP	1.0000	1.0000	1.0000	1.0000	1.0000	1.0000	1.0000
τ=0.95	SBP	1.0000	1.0000	1.0000	0.4034	1.0000	1.0000	1.0000
	DBP	1.0000	0.9986	1.0000	1.0000	1.0000	1.0000	0.9986

### 4.3. Model comparison

Observing at the 95% confidence intervals of frequentist approach and the 95% credible intervals of two Bayesian approaches from Tables 3 and 4, the BQRVS-FP model has tighter intervals compared to the QR-FP model having wider intervals.

Another finding is from the diagnostic plots that the autocorrelation plots of BQRVS-FP model have a faster decreasing rate across all the quantile levels, whereas those of the BQR-FP model have a slower rate. This is evident that the BQRVS-FP model has more random stationary posterior distributions of interest.

When looking at Tables 3–5, the BQRVS-FP model selected the important predictors coinciding with statistically significant associations between SBP, DBP and their risk factors based on their 95% credible intervals.

These findings suggest that the Bayesian variable selection approach to quantile regression model with FPs obtained more precise estimates than the frequentist and unregularised Bayesian approaches. The non-linear terms were selected as important variables in both SBP and DBP models indicating that FP model was necessary to examine the non-linear relationship between SBP, DBP and risk factors.

Whilst computational performance was not evaluated in this paper, it is noteworthy that all computations were executed on R version 4.2.2, utilising an Intel Core i7-4790 CPU@3.6GHz machine with 16GB DDR3 RAM memory. Both Rcpp and the Intel MKL compiler were employed to enhance the efficiency of the proposed method and reduce running time. The proposed method follows a three-stage algorithm, which, admittedly, demands more computational time compared to the unregularised Bayesian method that relies solely on a Gibbs sampling algorithm. Nevertheless, as previously mentioned, the second-stage algorithm of the proposed method, namely the Gibbs sampling algorithm, exhibits a faster convergence rate. Consequently, it necessitates fewer iterations to run compared to the unregularised Bayesian method. The first and last algorithms of the proposed method, requiring fewer iterations, contribute to a reasonable overall computational performance. It is crucial to note that, with an increasing amount of data, computational challenges may arise, potentially necessitating a big data strategy to address these issues. However, it is important to acknowledge that addressing these challenges extends beyond the scope of this paper.

## 5. Conclusion

In this paper, we conducted the data analysis of the impact of body mass index (BMI) on the blood pressure (BP) measures, including systolic and diastolic BP using data extracted from the 2007 to 2008 National Health and Nutrition Examination Survey (NHANES). The descriptive analysis showed that the prevalence of hypertension increased by age and the hypertension was highly prevalent amongst very obese and morbidly obese participants. In particular, it was more prevalent in men than women. Moreover, there was a statistically significant moderate association between SBP and age based on the Cramér's V value, whilst the remaining associations were weaker for both BP measures. However, there was no association between DBP and marital status.

The analysis motivated a new Bayesian non-linear quantile regression model under fractional polynomial (FP) model and variable selection with quantile-dependent prior. The quantile regression analysis investigates how the relationships differ across the median and upper quantile levels. The use of FPs allows for the relationships to be non-linear parametrically. The variable selection investigates for important predictors that contribute to the non-linear relationships via the Bayesian paradigm. The model analysis suggested that the proposed model provides better estimates because the 95% credible intervals were narrower and the autocorrelation plots have faster decreasing rates of correlated posterior samples in comparison to two methods, the frequentist and Bayesian approaches of quantile regression model. The analysis of the data showed that non-linear relations do exist because the proposed model identified the non-linear terms of continuous variables, including BMI and age as important predictors in the model across all the quantile levels. On the other hand, the non-linear term of age was not selected under the SBP model. The marital status was not selected as an important risk factor for the DBP model at the median level. This agreed with findings of both descriptive and model analyses. Moreover, the data analysis suggested that the quantile-based FP approaches have the goodness of fit in comparison to mean-based FP approaches. Thus, the importance of the non-linear quantile model with FPs is significant for modelling of BP measures.

We thanked the two referees and an associate editor for their thoughtful comments and suggestions, which have subsequently improved the quality of the manuscript.
